# In vitro and in situ study on characterization and mechanism of the intestinal absorption of 2,3,5,4′-tetrahydroxy-stilbene-2-O-β-D-glucoside

**DOI:** 10.1186/s40360-020-0384-9

**Published:** 2020-01-22

**Authors:** Cheng Wang, Yimeng Zhou, Xiaohong Gong, Li Zheng, Yunxia Li

**Affiliations:** 1 School of Pharmacy, Chengdu University of Traditional Chinese Medicine, Chengdu, 611137 China; 2 Key Laboratory of Standardization for Chinese Herbal Medicine, Ministry of Education, Chengdu, 611137 China; 3 National Key Laboratory Breeding Base of Systematic Research, Development and Utilization of Chinese Medicine Resources, Chengdu, 611137 China

**Keywords:** 2,3,5,4′-tetrahydroxystilbence-2-O-β-D-glucoside, Absorption mechanism, Caco-2 cell, Intestinal perfusion, P-gp, MRP2

## Abstract

**Background:**

2,3,5,4′-tetrahydroxystilbence-2-O-β-D-glucoside (TSG) is a polyhydroxyphenolic compound, which exhibited a broad spectrum of pharmacological activities, such as anti-inflammatory, anti-depression, anti-oxidation and anti-atherosclerosis. However, the compound had poor bioavailability and the underlying absorption mechanisms had not been studied. Therefore, the purpose of this study was to investigate the intestinal absorption mechanism of TSG.

**Methods:**

This study used Caco-2 cell monolayer model and single-pass intestinal perfusion model to explore the gastrointestinal absorption mechanisms of TSG. The effects of basic parameters such as drug concentration, time and pH on the intestinal absorption of TSG were analyzed by high performance liquid chromatography. The absorption susceptibility of TSG to three inhibitors, P-gp inhibitors verapamil hydrochloride and quinidine, and MRP2 inhibitor probenecid were also assessed.

**Results:**

TSG was poorly absorbed in the intestines and the absorption of TSG in stomach is much higher than that in intestine. Both in vitro and in situ experiments showed that the absorption of TSG was saturated with increasing concentration and it was better absorbed in a weakly acidic environment pH 6.4. Moreover, TSG interacts with P-gp and MRP2, and TSG was not only the substrate of the P-gp and MRP2, but also affected the expression of P-gp and MRP2.

**Conclusions:**

It was concluded that the gastrointestinal absorption the most unique active ingredient and considered as the mechanisms of TSG involved processes passive transport and the participation of efflux transporters.

## Background

**Fig. 1:**
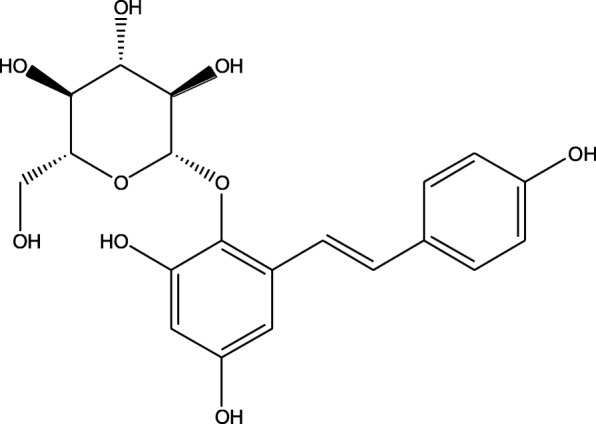
Chemical structure of TSG

*Polygonum multiflorum*, the dry root of *Polygonum multiflorum* Thunb. (Polygonaceae), is wildly used as a nourishing Chinese medicine for the remarkable pharmacological effects of neuroprotection, anti-oxidation, improving immunity, hypolipidemic, anti-atherosclerosis [1], anti-liver injury [2] and anti-cancer [3]. The complex chemical constituents of *Polygonum multiflorum* include stilbene glycosides, terpenoids, flavonoids, tannins, sugars and trace elements etc. Among them, 2,3,5,4′-tetrahydroxystilbene-2-O-β-D-glucoside (TSG) (Fig. 1) is the most unique active ingredient and considered as the quality index of *Polygonum multiflorum* in the 2015 edition of Chinese pharmacopoeia. It is stipulated that the content of TSG in raw and prepared *Polygonum multiflorum* should not be lower than 1.00 and 0.70% respectively. Modern studies have shown that TSG has a wide range of pharmacological effects, including anti-inflammatory [4], anti-depression [5], anti-oxidation [6], anti-atherosclerosis [7], improving gastrointestinal function [8] and protecting the cardiovascular system, etc. [9]. Clinically, TSG is used to prevent and treat hyperlipidemia [10], atherosclerosis [11], Alzheimer’s disease [12, 13], Parkinson’s disease [14, 15] and cerebral ischemia/reperfusion injury, etc. [16].

For most oral preparations, absorption into the blood circulation system is the prerequisite for drug efficacy. In previous study, our laboratory has conducted pharmacokinetic investigation on TSG after oral administration. The results showed that the poor bioavailability of TSG, indicating that only a small amount of TSG entered the blood circulatory system. This discovery led us to have a strong interest in the absorption and metabolism of TSG in the gastrointestinal tract.

In order to reveal the influencing factors and mechanisms of oral drug absorption, researchers have established a variety of models, and the commonly used methods are Caco-2 cell model, in situ intestinal perfusion model method, intestinal vascular intubation method and brush border membrane vesicle method [17, 18]. Caco-2 cells are internationally recognized as a classic in vitro model for studying the absorption properties and transport mechanisms of oral drugs [19, 20] because of their microvilli structure, biochemical properties similar to intestinal epithelial cells and enzymes associated with the intestinal brush epithelium, and expressing various transport proteins such as P-glycoprotein (P-gp) and multidrug resistance-associated protein 2 (MRP2). In situ intestinal perfusion method is characterized by simple operation, mature technology and strong controllability, which can ensure the integrity of intestinal neuroendocrine regulation and the blood supply of lymph fluid [21]. It is also widely used to study the absorption of drugs in the intestine.

Therefore, this study combines Caco-2 cell monolayer model and single-pass intestinal perfusion model to study the factors affecting the absorption of TSG in intestine. In addition, in order to investigate the intestinal transport mechanism of TSG, western blotting was carried out to explore the effect of TSG on P-gp and MRP2 expression during absorption by administering P-gp inhibitors (verapamil hydrochloride and quinidine) and MRP2 inhibitor (probenecid). We hope the study will provide a reference for improving the bioavailability of TSG, designing a reasonable dosing regimen and predicting drug interactions.

## Methods

### Laboratory reagents

2,3,5,4′-tetrahydroxystilbence-2-O-β-D-glucoside (TSG, purity ≥98.72%), rhaponticin (internal standard, IS, purity ≥98%), were obtained from Chengdu Chroma-Biotechnology Co., Ltd. Verapamil hydrochloride (purity ≥98%), quinidine (purity ≥98%), probenecid (purity ≥98%), were purchased from Chengdu Chroma-Biotechnology Co., Ltd. BCA protein quantification kit was purchased from Sichuan Sainst Biotechnology Co., Ltd. Anti-P glycoprotien antibody, Anti-MRP2 antibody, were purchased from Abcam, USA. Phenol red, GAPDH polyclonal antibody, purchased from Multisciences (Lianke) Biotech, co., Ltd. Ultrapure water (prepared by Yupu ultrapure water manufacturing system), HPLC-grade methanol, Phenol Red, acetonitrile, and formic acid (Fisher, USA).

### Experimental instruments

Agilent 1260 High Performance Liquid Chromatograph (Agilent, USA); Flow Type Intelligent Peristaltic Pump (BT101L/G, Baoding Reef Fluid Technology Co., Ltd.); Electronic analytical balance (Sartorius 11D, Sartorius, Germany); Xiangyi desktop high-speed centrifuge (TG16-WS, Hunan Xiangyi Laboratory Instrument Development Co., Ltd.); Ultrapure water meter (TANKPE030, Sichuan Yourun Technology Co., Ltd.); Carbon dioxide incubator (Forma 3111, Thermoscientific, USA); Purification workbench (SW-CJ-2FD, Suzhou Antai Air Technology Co., Ltd. Company); Ultrasonic Cell Crusher (SCIENTZ-IID, Ningbo Xinzhi Biotechnology Co., Ltd.); 12-well Polycarbonate Membrane Transfer Plate (3460 Transwell, Costar, USA, 0.4 μm pore size, 12 mm diameter, bottom area 1.12 cm^2^); Electronic Constant Temperature Water Bath (DZKW-4, Beijing Zhongxing Weiye Instrument Co., Ltd.).

### Caco-2 cell monolayer model

#### Cytotoxicity of TSG for Caco-2 cells

MTT assay was firstly used to investigate the effect of TSG with different concentrations on Caco-2 cells vitality. Briefly, Caco-2 cells were seeded in 96-wells plates at a density of 1 × 10^4^ cells per well and placed in a 5% carbon dioxide incubator. After 48 h incubation, the cells were treated with increasing concentrations of TSG (0.75–240 μg·mL^− 1^) and DMEM (containing 1%FBS) as a negative control. After 5 h of incubation, 20 μL of 5 mg·mL^− 1^ MTT was added to each well, and the plate was incubated at 37 °C for 4 h. Then 150 μL of DMSO was added to each well after the supernatant was discarded. Absorbance was measured at a wavelength of 570 nm.

#### Caco-2 cell culture

Caco-2 cells (purchased from the Shanghai Cell Bank of the Chinese Academy of Sciences, 35 to 50 generations) were routinely cultured in DMEM containing 20% FBS, 1% nonessential amino acids, 1% penicillin and 1% streptomycin at 37 °C in a 5% carbon dioxide incubator. When the cells grew to 80–90%, 0.5 mL of the well-mixed Caco-2 cell suspension at density of 1 × 10^5^ cells·mL^− 1^ was added to AP side of 12-well polycarbonate membrane transfer plate. On the 1st to 7th day of cell growth, the medium of AP and BL side were replaced with fresh DMEM every other day. On the 8th to 14th day, the DMEM were changed every other day and only the AP side was replaced on even days. After 15th day, the medium of both chambers was changed every day. After 21 days of standardized culture, the morphology of Caco-2 cell monolayer was observed by electron microscopy and the cell monolayers with a TEER above 500 Ω·cm^− 2^ were selected for transport assays. The membrane integrity and transport function of Caco-2 cell monolayer model were further verified with propranolol hydrochloride and atenolol.

#### Absorption and transportion of TSG on Caco-2 cell monolayer

37 °C HBSS was used as the buffer for bidirectional transport including absorption transport from AP to BL side and secretion transport from BL to AP side. Before transport studies, the cell monolayer was washed three times with 37 °C HBSS. In the transport experiment from AP side to BL side, 0.5 mL of TSG solution at a concentration of 10, 30 or 60 μg·mL^− 1^ was added to AP side and 1.5 mL of HBSS was added to BL side. On the contrary, in the transport experiment from BL side to AP side, 1.5 mL TSG solution at a concentration of 10, 30 or 60 μg·mL^− 1^ was added to BL side and 0.5 mL of HBSS was added to AP side. After administration, the transfer plate was placed in an incubator. 0.3 mL sample was taken on BL side and 0.1 mL samples was taken on the AP side at different time points (0.5, 1.0, 1.5, 2.0, 2.5, 3.0, 3.5 and 4.0 h). After each sampling, an equal volume of HBSS was added to the receiver chamber to maintain a constant volume. In addition, we also explored the transportation of TSG at different pH conditions (5.4, 6.4 and 7.4). The pH of DMEM (containing 1% FBS) was adjusted to 5.4 and 6.4, respectively*,* and TSG solutions of different pH were prepared for transport experiments to investigate the transport of TSG at different pH conditions.

#### Effects of P-gp and MRP2 on the absorption of TSG

The effect of efflux protein inhibitors on the intestinal absorption of TSG was studied based on Caco-2 cell monolayer model. An appropriate amount of verapamil hydrochloride, quinidine and probenecid were accurately weighed and dissolved in DMEM to obtain an inhibitor solution with a concentration of 5 and 25 μg·mL^− 1^. Before the administration, the inhibitor was added to AP side and BL side, and the plate was placed in an incubator. After 30 min, the inhibitor solution was replaced with TSG solution at concentration of 30 μg·mL^− 1^ to inspect the effect of efflux proteins on TSG.

#### Effect of TSG on the expression of P-gp and MRP-2

When Caco-2 cells grew to 80–90%, the cell were treated with different concentrations (10, 30 and 60 μg·mL^− 1^) of TSG and blank medium solution (blank control group). After 4 h of incubation, the cell was treated with lysates, homogenized on ice for 10 min, centrifuged at 12000 r·min^− 1^ at 4 °C for 10 min, and then the supernatant was harvested. The concentration of total proteins was detected by BCA kit, and the supernatant were mix with SDS-PAGE sample buffer after protein quantification, denatured on a heater for 5 min and stored.

The total proteins were electrophoresed in 10% SDS-PAGE gel and then transferred to PVDF membranes by wet process to block with 5% skim milk for 2 h at room temperature. Membranes were incubated overnight with the following primary antibodies Anti-P glycoprotein antibody (1:2000), Anti-MRP2 antibody (1:500), and GAPDH polyclonal antibody. After that, the membranes were washed with 0.1% TBST for 3 times and 10 min each time and incubated with corresponding secondary antibody (1:5000) for 1 h at room temperature. The OD density values of bands were analyzed using gel imaging and analysis system.

#### Determination of TSG by HPLC

The samples were centrifuged at 12000 r·min^−^ ^1^ at 4 °C for 15 min. Then, the supernatant was analyzed by HPLC. HPLC separation was performed on Eclipse Plus C_18_ column (4.6 mm × 250 mm, 5 μm) at 30 °C. The mobile phase consisted of acetonitrile and 0.1% formic acid water (25:75) and the velocity of flow is 1 mL·min^− 1^. The injected sample volume was 5 μL. TSG was detected at wavelength of 320 nm.

### The intestinal perfusion in situ model

#### Experimental animal

Sprague-Dawley rats (200 ± 20 g) were supplied by the Animal Center of Chengdu University of TCM. All the rats were housed in a standard animal laboratory (23 ± 2 °C, relative humidity 50 ± 20%) with a 12 h light/dark cycle. Food was prohibited for 12 h before the experiment while water was provided freely. The animal experiments were conducted in accordance with Guide for the Care and Use of Laboratory Animals (NIH publication #85–23, revised in 1985). The Animal Ethics Committee of Chengdu University of Traditional Chinese Medicine granted the experiment SCXK 2013–19.

#### Preparation of test solution

##### Preparation of artificial gastric juice

According to the Chinese Pharmacopoeia, the artificial gastric juice without pepsin is configured under the time limit inspection method of disintegration.

##### Preparation of gastric perfusate

The appropriate amount of TSG was accurately weighed, and the gastric perfusate was prepared at a concentration of 10, 30 and 60 μg·mL^− 1^ using artificial gastric juice.

##### Preparation of Krebs-ringer (K-R) buffer

Weigh MgCl_2_: 0.02 g, NaCl: 7.80 g, KCl: 0.35 g, NaHCO_3_: 1.37 g, NaH_2_PO4: 0.32 g, in 1000 mL volumetric flask, add pure water to dissolve it fully, then 0.37 g of CaCl_2_ was added while ultrasonication, and 1.40 g of Glucose was added at the time of use, sonicated, and the volume was adjusted with pure water to determine the pH of 7.4. All buffer solutions were prepared and used on the same day.

##### Preparation of blank intestinal circulating fluid

Accurately weigh the appropriate amount of phenol red, dissolve it with K-R buffer and dilute it into a phenol red solution with a mass concentration of 20 μg·mL^− 1^.

##### Preparation of intestinal circulation fluid

The proper amount of TSG was accurately weighed, and the intestinal circulation liquid with a concentration of 10, 30 and 60 μg·mL^− 1^ was prepared by using a blank intestinal circulation solution.

#### In situ single-pass intestinal perfusion studies in rats

24 SD rats, half male and half female, were fasted for 12 h but permitted to drink water freely before surgery. Rats were anesthetized by intraperitoneal injection of 25% urethane (0.05 mL·g^− 1^) and placed in a supine position on the operating table. The abdominal cavity was opened along the midline of the abdomen, then the incision was made at the duodenum and the ileum. The polyethylene tubes were carefully inserted at both sides of the segment and ligatured with a sterile surgical line. The contents of the intestines were slowly rinsed with 0.9% saline, prewarmed at 37 °C, and the residual liquid in the intestine was drained with air. Afterward, the wound in the abdomen of the rats was covered with a gauze soaked with 37 °C 0.9% saline buffer to moisturize after the operation, warmed with infrared lamp. The intestinal was equilibrated with a drug-containing perfusate (the concentration of TSG was 10, 30 and 60 μg·mL^− 1^ respectively) which was preheated at 37 °C at a flow rate of 5 mL·min^− 1^. Samples were collected in EP tubes after 10 min, and blank intestinal circulation solution (1 mL) was quickly added to circulatory solution. After that, the flow rate was adjusted to 2 mL·min^− 1^, and the samples were taken at 10 min intervals for 4 h. In addition, in order to study the effects of different pH on intestinal absorption of TSG, the pH of the blank intestinal circulation solution was adjusted to 5.4 and 6.4, respectively. Finally, the experimental animals were euthanized by the method of rapid decapitation when the experiment was over. The procedures and precautions for sacrifice were the same as previously described.

#### Effects of P-gp and MRP2 on the absorption of TSG

The effect of efflux protein inhibitors on the intestinal absorption of TSG was studied based on the intestinal recirculating perfusion model. The verapamil hydrochloride, quinidine and probenecid were dissolved in blank intestinal circulation solution to obtain an inhibitor solution of 5 and 25 μg·mL^− 1^. The perfusion was started with inhibitor solution at a flow rate of 2 mL·min^− 1^, and after 30 min, it was replaced with TSG solution to investigate the influences of efflux proteins on TSG.

#### Determination of TSG by HPLC

1 mL sample was mixed with equal amount of methanol, filtered through a membrane filter (0.45 μm pore size), and centrifuged at 12000 r·min^− 1^ for 10 min. The concentration of TSG and phenol red of supernatant were measured by HPLC on Eclipse Plus C_18_ column (4.6 mm × 250 mm, 5 μm) at 30 °C. The mobile phase consisted of (A) 0.1% formic acid water and (B) acetonitrile. The gradient elution was set as: linear gradient elution 20–35% B (0-15 min) with a flow rate of 1 mL·min^− 1^. The injected sample volume was 5 μL. TSG was detected at wavelength of 320 nm and phenol red was detected at wavelength of 430 nm. The concentration of phenol red was used to correct the volume of intestinal circulating fluid. The concentration of TSG and the volume of intestinal circulating fluid in each time period was used to calculate the residual drug amount (X). With the sampling time t as independent variable and lnX as dependent variable, the absorption rate constant Ka was determined from the slope of the straight line. The drug absorption percentage were calculated by the ratio of the change value of the remaining dose of t (h) to the amount of the remaining drug at time 0.

### Data analysis

The effective permeability coefficient (P_app_, cm/s) across Caco-2 cell monolayer was calculated from the linear plot of drugs accumulated in the receiver side versus time using the following eq. (1):

1 $$ {\mathrm{P}}_{\mathrm{app}}=\mathrm{dQ}/\mathrm{dt}\times 1/\mathrm{A}\times 1/{\mathrm{C}}_0 $$

Where dQ/dt represented the steady-state flux of the drug on the receiver side, C_0_ was the initial concentration of the compound in the donor side, and A was the surface area of the polycarbonate membrane.

Since the HBSS buffer was added after each sampling to dilute the drug concentration, the cumulative absorption concentration (C_cum_) of the drug was corrected using the following eq. (2):

2 $$ {\mathrm{C}}_{\mathrm{cum}}={\mathrm{A}}_{\mathrm{n}}+\frac{{\mathrm{V}}_{\mathrm{n}}}{\mathrm{V}}{\sum}_{\mathrm{i}=0}^{\mathrm{n}-1}{\mathrm{A}}_{\mathrm{i}} $$

where C_cum_ represented the cumulative concentration of the drug, A_n_ represented the permeation concentration of the nth sample, V_n_ represented the sampling volume of the nth sample, and V represented the volume of the receiving pool.

The absorption rate constants (Ka) and percentage of absorption (PA, %) were calculated using the following eqs. (3), (4), respectively.

3 $$ \mathrm{lnX}={\mathrm{lnX}}_0-\mathrm{Ka}\cdotp \mathrm{t} $$

4 $$ \mathrm{PA}=\left({\mathrm{C}}_0{\mathrm{V}}_0-{\mathrm{C}}_{\mathrm{t}}{\mathrm{V}}_{\mathrm{t}}\right)/{\mathrm{C}}_0{\mathrm{V}}_0\mathrm{t}\times 100\% $$

Where X was the residual drug amount, C_0_ was the drug concentration of intestinal circulation liquid at 0 min, V_0_ was the drug volume of intestinal circulation liquid at 0 min, C_t_ was the drug concentration of intestinal circulation liquid at t min, V_t_ was the drug volume of intestinal circulation liquid at t min, t was the perfusion time.

### Statistical analysis

All experiments results were expressed as the mean ± SD. Statistical comparisons were performed by one-way analysis of variance (ANOVA) using SPSS 21.0. When *p* < 0.05, it was considered statistically significant.

## Results

### Caco-2 cell monolayer model

#### The results of the cytotoxicity test

**Fig. 2:**
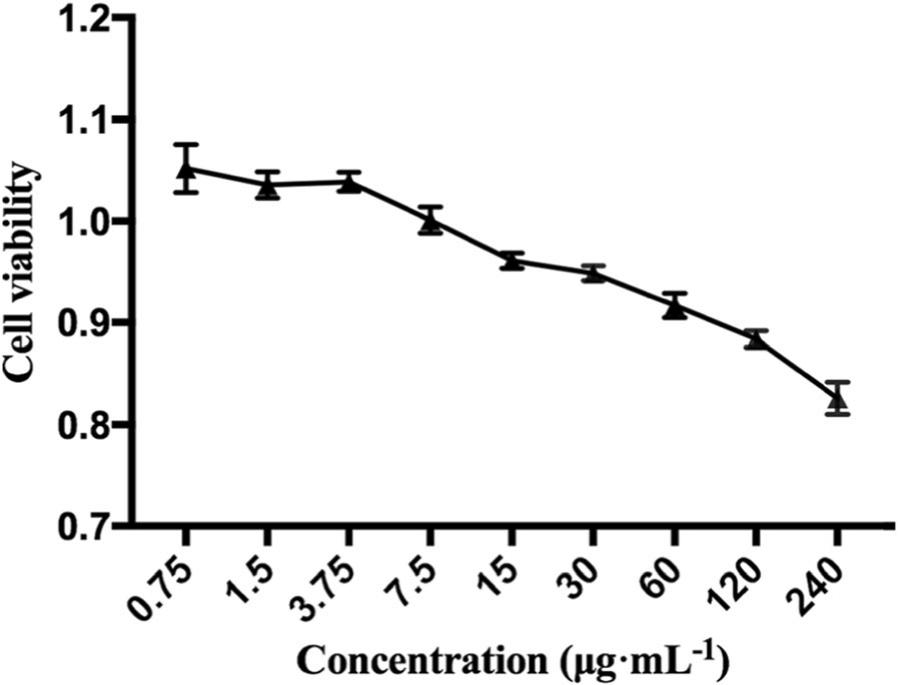
Cell viability of TSG on Caco-2 cells (*n* = 3)

In order to avoid the false positive result of the target drug on the cells, we examined the effect of the target drug on cell viability by MTT assay before the transport experiment. The results are shown in Fig. 2. The cell viability in the presence of 60 μg·mL^− 1^ TSG was greater than 90%. When the concentration of TSG was more than 60 μg·mL^− 1^, the cell viability was significantly decreased. Therefore, transportation studies were conducted at concentrations of 10, 30, and 60 μg·mL^− 1^ TSG.

**Fig. 3:**
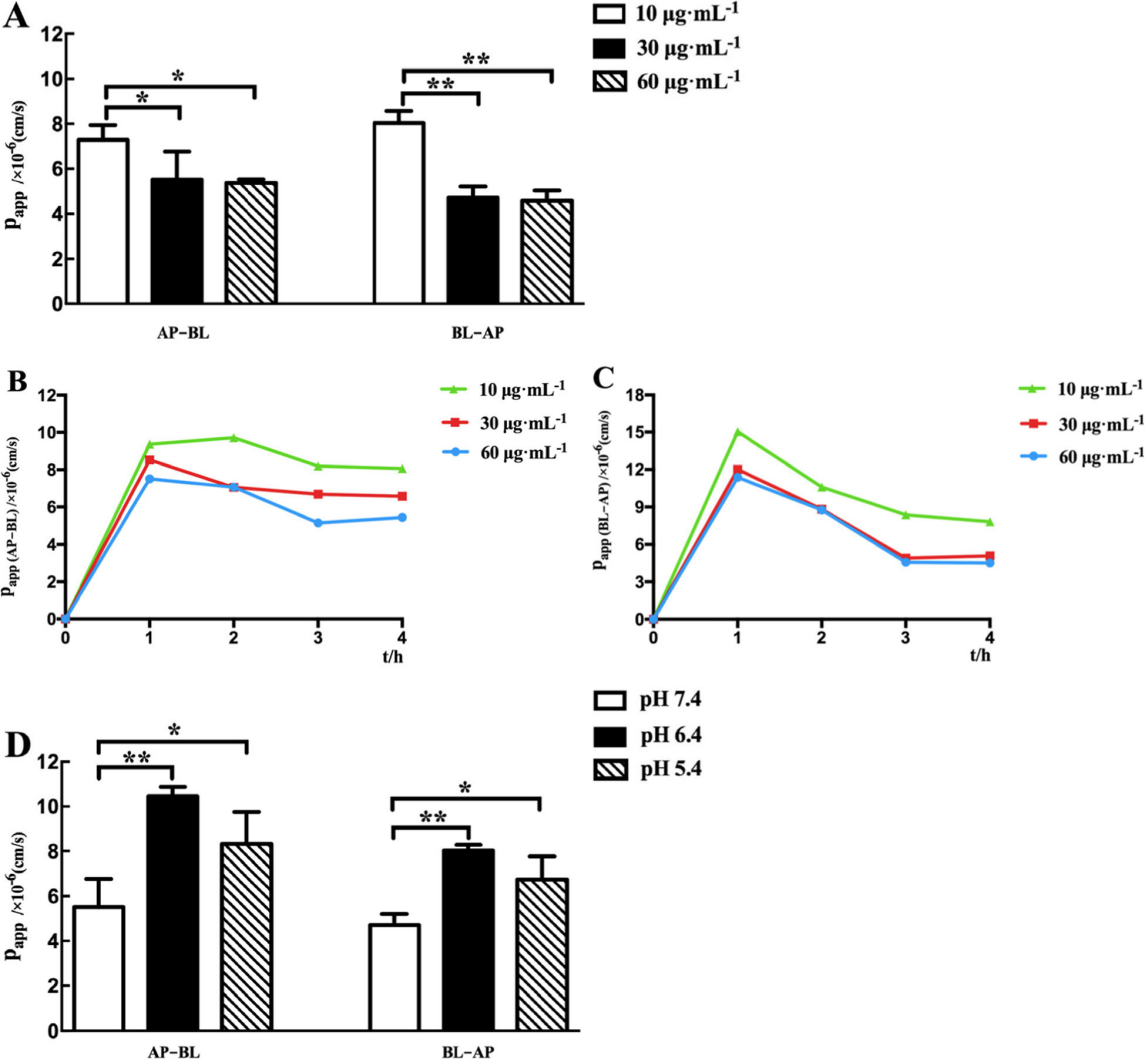
Characterization of the intestinal permeability features of TSG in Caco-2 cell monolayer. **a** Bidirectional transport studies of TSG at different drug concentrations (10, 30 and 60 μg·mL^− 1^, respectively). *p* < 0.05 (*), *p* < 0.01 (**), comparison with the group at 10 μg·mL^− 1^ TSG. **b** The apparent permeability (P_app_, cm/s) values of AP-BL at 1, 2, 3 and 4 h for TSG. **c** The apparent permeability (P_app_, cm/s) values of BL-AP at 1, 2, 3 and 4 h for TSG. **d** The P_app_ of TSG at different pH values (7.4, 6.4 and 5.4, respectively). *p* < 0.05 (*), *p* < 0.01 (**), comparison with the pH 7.4 group. All results are expressed as mean ± SD (*n* = 3)

**Fig. 4:**
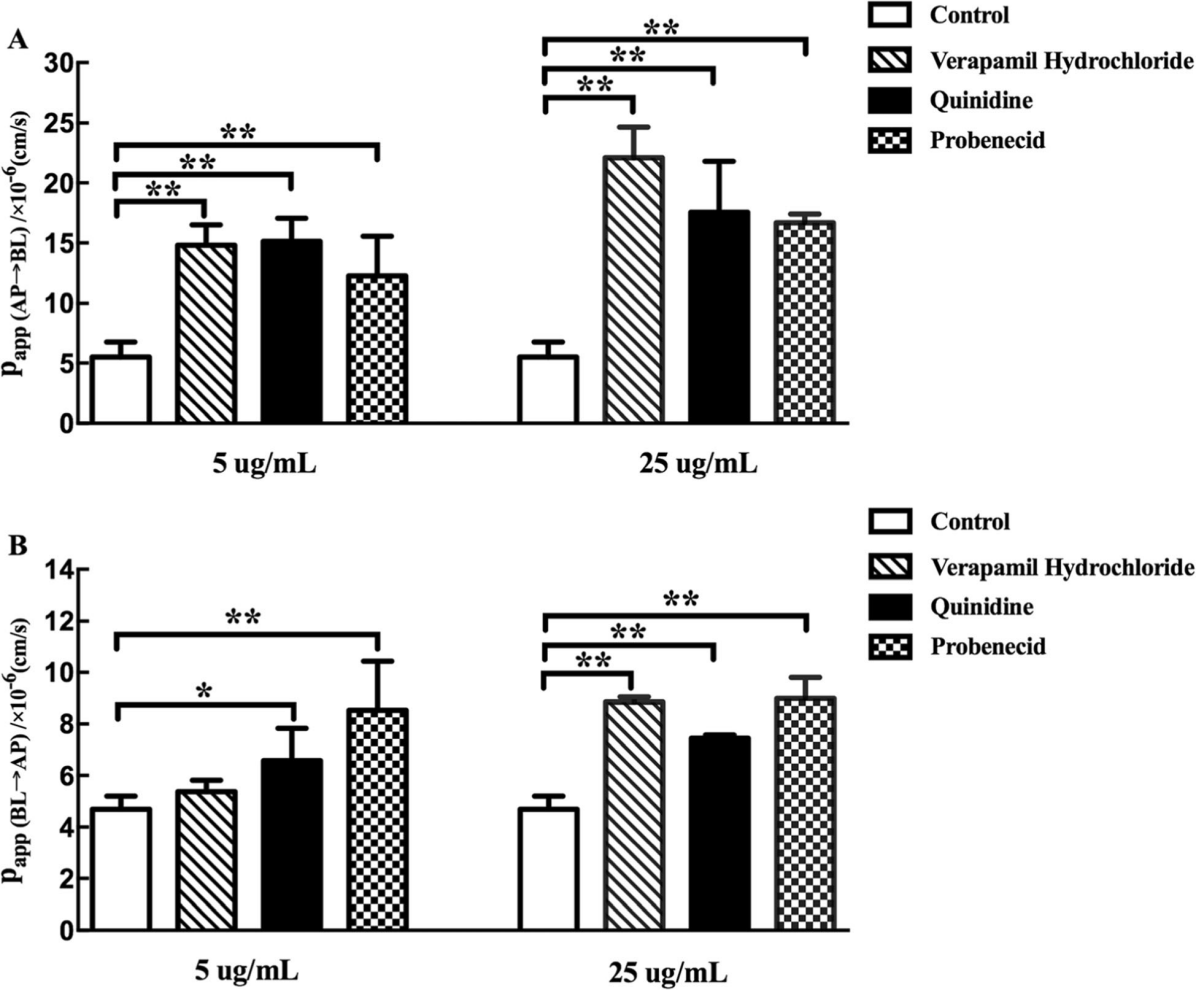
Effect of different inhibitors on the transport of TSG across Caco-2 cell monolayer. The data are presented as the apparent permeability (P_app_, cm/s). Effect of P-glycoprotein (P-gp) inhibitor (verapamil hydrochloride and probenecid) and multidrug resistance-associated protein 2 (MRP2) inhibitor (probenecid) on Caco-2 cell monolayer for TSG. AP-BL side (**a**); BL-AP side (**b**). *p* < 0.05 (*), *p* < 0.01 (**), comparison with control. All results are expressed as mean ± SD (*n* = 3)

**Fig. 5:**
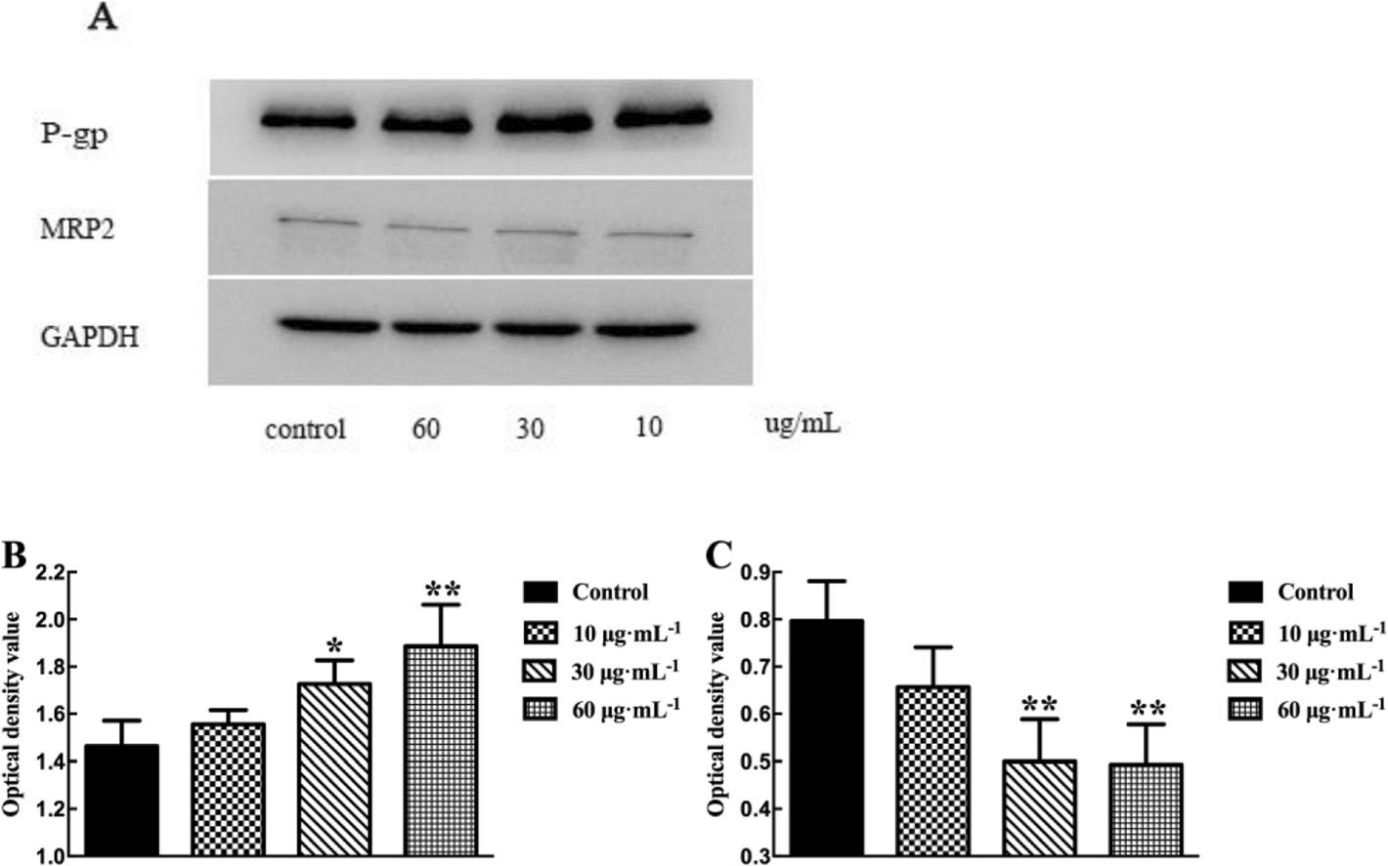
Effects of TSG on the expression of P-gp and MRP2 in Caco-2 cell monolayer. **a** The effects of TSG on the expression of P-gp and MRP2. **b** The expression of P-gp in Caco-2 cell after treat with TSG. **c** The expression of MRP2 in Caco-2 cell after treat with TSG. *p* < 0.05 (*), *p* < 0.01 (**), comparison with control. All results are expressed as mean ± SD (*n* = 3)

**Fig. 6:**
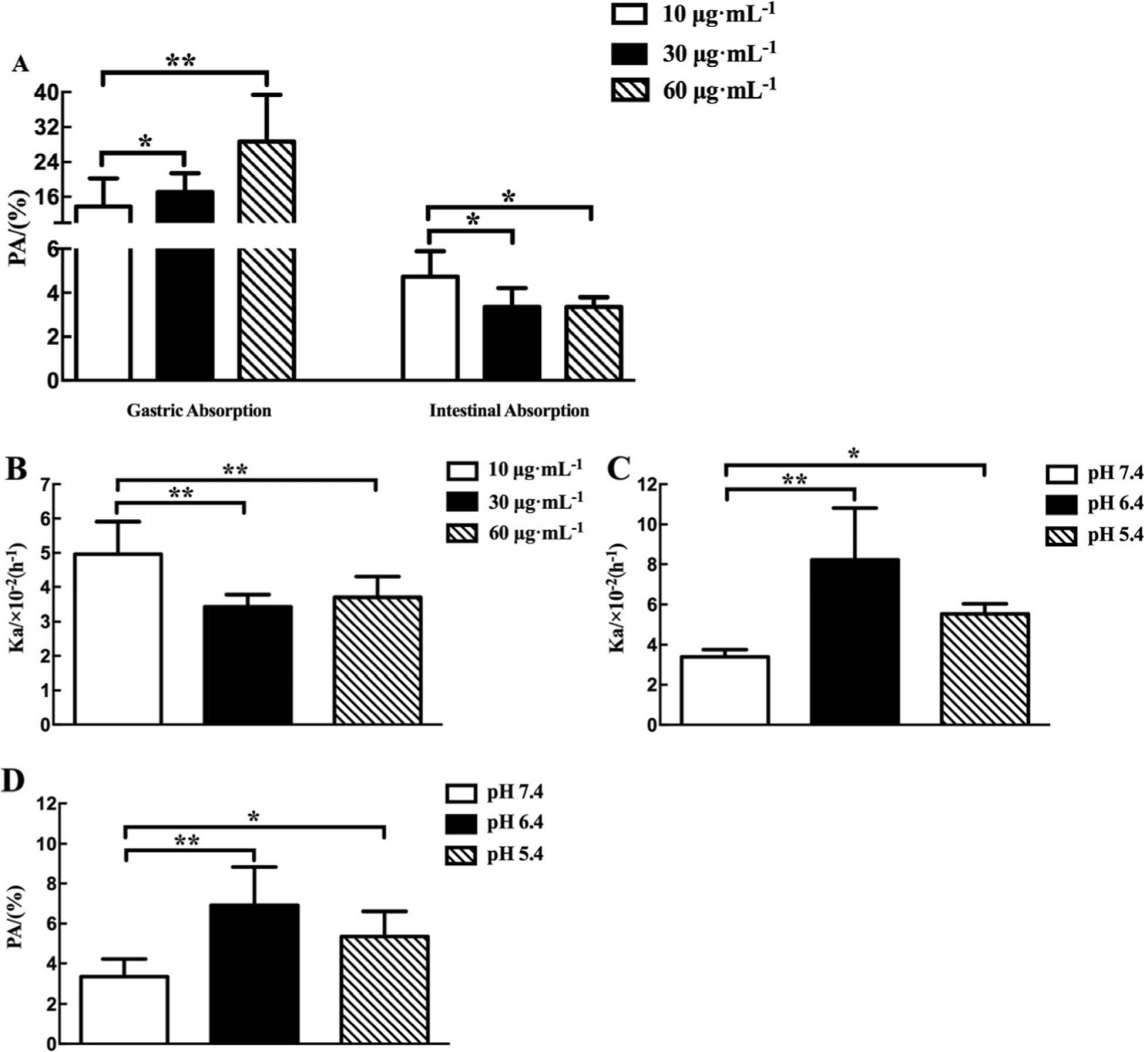
Characterization of the intestinal permeability features of TSG in single-pass intestinal perfusion model. The data are presented as absorption rate constants (Ka, h^− 1^) values and percentage of absorption (PA, %) values. **a** Absorption of TSG in the stomach and small intestine. **b** The absorption rate constants (Ka, h^− 1^) values of TSG at different drug concentrations. *p* < 0.01 (**), comparison with the group at 10 μg·mL^− 1^ TSG. **c** The absorption rate constants (Ka, h^− 1^) values of TSG at different pH values. *p* < 0.05 (*), *p* < 0.01 (**), comparison with the group at pH 7.4. **d** The percentage of absorption (PA, %) values of TSG at different pH values. *p* < 0.05 (*), *p* < 0.01 (**), comparison with the group at pH 7.4. All results are expressed as mean ± SD (*n* = 6)

**Fig. 7:**
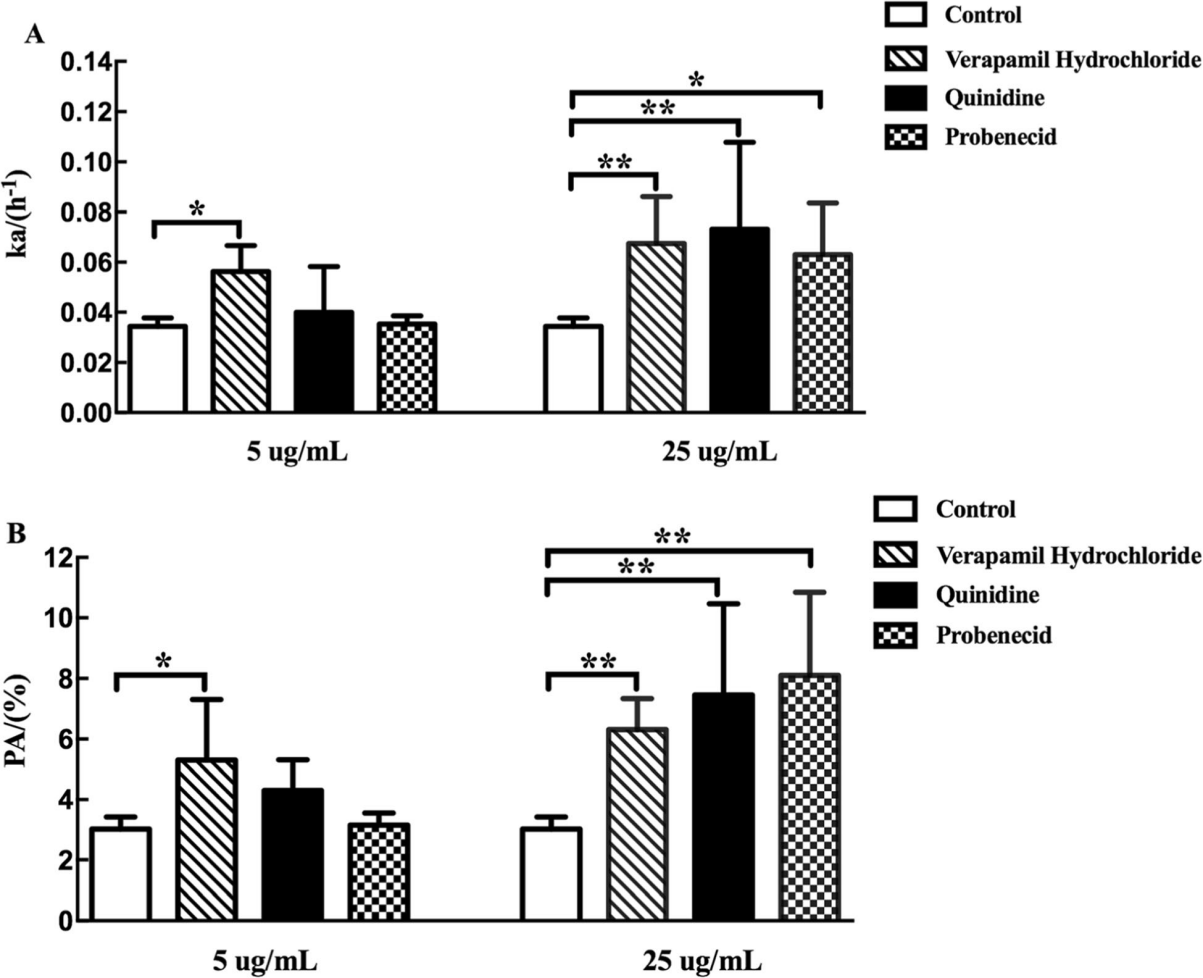
Effect of different inhibitors on intestinal permeability of TSG in rats. The data are presented as absorption rate constants (Ka, h^− 1^) values and percentage of absorption (PA, %) values. Effect of P-glycoprotein (P-gp) inhibitor (verapamil hydrochloride and probenecid) and multidrug resistance-associated protein 2 (MRP2) inhibitor (probenecid) on small intestinal absorption of TSG. Absorption rate constants (Ka, h^− 1^) values (**a**); Percentage of absorption (PA, %) values (**b**). *p* < 0.05 (*), *p* < 0.01 (**), comparison with control. All results are expressed as mean ± SD (*n* = 6)

#### The characterization of Caco-2 cell monolayer

The integrity of the Caco-2 cell monolayer was evaluated by measuring TEER and transport studies of propranolol hydrochloride and atenolol, an internationally recognized positive control. In the study, Caco-2 cell monolayer displayed a TEER value high than 500 Ω·cm^− 2^ after 21 days of culture in the transfer plate. Propranolol hydrochloride and atenololl showed different absorption in the Caco-2 cell monolayer model. The results of transport experiments showed that the P_app_ values of AP-BL of propranolol hydrochloride on this model was (1.56 ± 0.24) × 10^− 5^ cm·s^− 1^, and the P_app_ values of AP-BL of atenolol was (5.82 ± 1.25) × 10^− 7^ cm·s^− 1^, which were in a good agreement with the P_app_ reported in the literature. These results indicated that Caco-2 cell monolayer model has tight junction and transport capacity and can be used for transport experiments.

#### The characterization of the intestinal permeability features of TSG

Caco-2 cell monolayer model was used to explore the intestinal permeability features of TSG. Firstly, the P_app_ values were measured at different TSG concentrations (10, 30 and 60 μg·mL^− 1^). It was found that the P_app_ values of TSG were between 1 × 10^− 6^ and 10 × 10^− 6^ cm/s, which was also between propranolol hydrochloride and atenolol. The result indicated that TSG was a moderately absorbed drug. As shown in Fig. 3a, the bilateral (AP-BL and BL-AP) P_app_ of TSG decreased substantially with increasing concentration and remained basically unchanged after reaching a certain concentration. In the bidirectional transport experiment, there were significant difference between low and medium concentrations, low and high concentrations (*p* < 0.05). However, no significant difference was found between medium concentrations and high concentrations of TSG (*p >* 0.5). Furthermore, the absorption of TSG on Caco-2 cell monolayer model did not have time-dependent relation which was indicated by the P_app_ values obtained after incubation with TSG for 1, 2, 3 and 4 h across Caco-2 cell monolayer (Fig. 3b and Fig. 3c). The bilateral P_app_ values of different concentrations of TSG reached the maximum at 1 h after administration, and gradually decreased to a steady state with the prolongation of time. The rapidity and saturation of absorption indicated that the transportation mechanism of TSG may involve some carrier protein.

Subsequently, the pH of DMEM (containing 1% FBS) was adjusted to 6.4 and 5.4, respectively. The effects of different pH values on TSG transport at a concentration of 30 μg·mL^− 1^ were shown in Fig. 3d. P_app_ values of TSG bidirectional transport increased significantly with the decrease of pH value. The bidirectional P_app_ value of TSG at pH 6.4 and 5.4 were significantly higher than that of pH 7.4 (*p* < 0.05). However, there was no significant difference between the P_app_ value of pH 6.4 and pH 5.4 (*p >* 0.05). Our data demonstrated that the transportation of TSG may be affected by acidic conditions and pH 6.4 was more suitable for the absorption of TSG.

#### The role of P-gp and MRP2 on TSG transport across Caco-2 cell monolayer

The role of efflux transporter on the permeability of TSG was also investigated in Caco-2 cell monolayer, and the results were showed in Fig. 4. Compared with TSG alone, 5 μg·mL^− 1^ P-gp inhibitor (verapamil hydrochloride and quinidine) and MRP2 inhibitor (probenecid) increased the P_app_ values of TSG about 2.68 times (*p* < 0.01), 2.74 times (*p* < 0.01) and 2.22 times (*p* < 0.01) on AP-BL side and 1.14 times (*p >* 0.05) 1.26 times (*p* < 0.05) and 1.82 times (*p* < 0.01) on BL-AP side. Similarly, the high dosage of 25 μg·mL^− 1^ P-gp inhibitor (verapamil hydrochloride and quinidine) and MRP2 inhibitor (probenecid) increased the P_app_ values 1about 4 times (*p* < 0.01), 3.18 times (*p* < 0.01) and 3.02 times (*p* < 0.01) on AP-BL side and 1.88 times (*p* < 0.01), 1.51 times (*p* < 0.01) and 1.91 times (*p* < 0.01), respectively on BL-AP side. The increased absorption by verapamil hydrochloride, quinidine and probenecid suggested that P-gp and MRP2 mediated the transport of TSG.

#### Effects of TSG on the expression of P-gp and MRP2 in Caco-2 cell monolayer

The results of western blotting experiments were shown in Fig. 5. After incubation with different concentrations of TSG (10, 30 and 60 μg·mL^− 1^), the expression of P-gp in Caco-2 cells increased with concentration and significant difference was observed at 60 μg·mL^− 1^ (*p* < 0.05). However, the expression of MRP2 in cells was decreased, and the inhibitory effects of 30 and 60 μg·mL^− 1^ of TSG were significant (*p* < 0.05). Therefore, not only TSG transportation was medicated by P-gp and MRP2, the induce of P-gp expression and inhibition of MRP2 expression were also existed, which indicated an interaction between TSG and transporter.

### Single-pass intestinal perfusion in situ model

#### The characterization of the intestinal permeability features of TSG in rats

The single-pass intestinal perfusion model was used to further explore the intestinal permeability features of TSG in rats. Firstly, we detected the absorption of different concentrations (10, 30 and 60 μg·mL^− 1^) of TSG in stomach. These results showed that TSG was well absorbed in stomach, and the absorption increased significantly with concentration (*p* < 0.05), which showed a passive transport mechanism.

Subsequently, we measured the effect of concentration (10, 30 and 60 μg·mL^− 1^) on the percentage of TSG absorption (PA, %) and absorption rate constants (Ka, h^− 1^) values. As shown in Fig. 6a and Fig. 6b, The PA and Ka of TSG of 10 μg·mL^− 1^ were significantly greater than the drug concentration at 30 and 60 μg·mL^− 1^ (*p* < 0.05). However, there was no significant difference between medium and high concentrations (*p* > 0.05). The absorption of TSG remained unchanged after certain concentration in small intestine. Therefore, it was speculated that TSG was mainly absorbed into blood circulation system through carrier transport in in small intestine.

The Ka and PA values of TSG were measured at different pH values (7.4, 6.4 and 5.4). As shown in Fig. 6c and Fig. 6d, the Ka of TSG showed a significant increase between pH 6.4 and 7.4 (*p* < 0.01), and the PA value of pH 7.4 was significantly different from the other two groups (*p* < 0.05). In other words, TSG shows better absorption in a weakly acidic environment.

#### The role of P-gp and MRP2 on intestinal permeability of TSG

To further confirm the role of P-gp and MRP2 on the intestinal permeability of TSG, the Ka and PA values were measured in the presence of verapamil hydrochloride, quinidine and probenecid protein inhibitors (Fig. 7). The results showed that the Ka and PA values of TSG increased significantly (*p* < 0.05) with verapamil hydrochloride and quinidine (25 μg·mL^− 1^) which indicated the involvement of P-gp in the intestinal epithelium transportation of TSG. The Ka and PA values of TSG were also significantly enhanced by 25 μg·mL^− 1^ probenecid (*p* < 0.05) which showed that the intestinal permeability of TSG was limited by MRP2. In summary, the in situ transport data indicated that TSG may be the substrate of efflux protein P-gp and MRP2.

## Discussions

TSG, also named 2,3,5,4′-tetrahydroxystilbence-2-O-β-D-glucoside, is a unique active component of *Polygonum multiflorum*. As a quality control index specified in Chinese Pharmacopoeia, TSG possessed various effects including anti-aging, lowering blood lipids, anti-atherosclerosis, anti-tumor and liver protection [6–9]. Previously, studies have evaluated the pharmacokinetics, tissue uptake, distribution and excretion of TSG systematically, all of which indicated a poor bioavailability and low tissue exposure of TSG. For example, Zhao et al. [22] detected TSG concentration in rats plasma using LC-Q-TOF-MS. According to the fitting analysis of two-compartment model, the maximal concentration of TSG (C_max_) was 5.70 μg·mL^− 1^, indicating that only a small amount of TSG was absorbed, the absorption half-life (t_1/2_K_a_) was 14.80 min, indicating that TSG can be absorbed rapidly in vivo. The results were consistent with the conclusions of noncompartmental analysis of Lv et al. [23, 24]. However, few studies focused on the reason of low bioavailability of TSG, and the absorption mechanisms in the gastrointestinal tract needed be clarified. Therefore, this study aimed to explore the absorption characteristics and its potential mechanism of TSG by cell model and in situ method.

Caco-2 cell is an international recognized drug absorption model which is widely used in drug research and development, and it is reported that there is a certain correlation of transportation and absorption of drugs between in vitro and in situ [25]. In this study, we examined the effect of TSG on cell viability by MTT assay before the transport experiment, which helps to eliminate the influence of toxicity of TSG on the cells. MTT assay is a commonly used method for detecting cell growth, cell activity and cytotoxicity. In our study, the results of MTT assay showed that TSG had basically no toxicity on Caco-2 cells within the concentrations of 0.75–60 μg·mL^− 1^. Therefore, subsequent transport experiments were conducted at concentrations of 10, 30, and 60 μg·mL^− 1^ TSG.

In Caco-2 cell monolayer model, our data showed that the P_app_ value of TSG ranged from 1.0 × 10^− 6^ to 10.0 × 10^− 6^ cm·s^− 1^, which indicated TSG was absorbed moderately according to the internationally accepted standard. The results of cell transfer assay showed that the absorption of TSG in Caco-2 cell model did not have time-dependent relation, and the P_app_ values of bidirectional transport at different concentrations all reached maximum at 1 h. With the prolongation of time, the P_app_ values of TSG gradually decreased and tended to balance. It suggested that the transport of TSG in the intestine mainly relyed on carrier proteins. In addition, the different pH conditions influenced the transport of TSG with a better absorption in weakly acidic conditions at pH 6.4.

On the other hand, in single-pass intestinal perfusion model, the absorption of TSG in stomach was much higher than that of intestine. The increased absorption of TSG in stomach with concentrations showed a passive transport mechanism. However, the absorption of TSG in intestine decreased when concentration increased and showed a saturation when the concentration reached a certain value, which suggested the transportion invovling carrier proteins. The results were consistent with the transport experiments in vitro. Interestingly, we also found TSG showed a better absorption in the weakly acidic conditions at pH 6.4 in single-pass intestinal perfusion model. The reason may be that TSG contained multiple phenolic hydroxyl groups, which were mainly present in molecular form in the acidic conditions and had good lipid solubility. The absorption increased in the weakly acidic conditions at pH 6.4, which may account for the transport mechanism of passive diffusion.

The process of drug absorption in the body is complicated and is affected by many factors, such as the physical and chemical properties of drug, the gastrointestinal environment, and the transporter on the intestinal tract [26]. A variety of transporters are expressed on the membrane of intestinal epithelial cells, among which the outward transporter (efflux protein) can actively discharge drugs from cells, which lead to the reduction of drug absorption. In recent years, efflux protein-mediated drug efflux has attracted extensive attention. The efflux protein is an energy-dependent protein widely distributed in various tissues of the body mainly inhibiting transmembrane of drugs [27, 28]. P-gp and MRP2 are highly expressed in the brush border of intestinal mucosal epithelial cells and have been studied extensively at present. The expression of P-gp is gradually increased from the proximal to the distal of intestine, and MRP2 is expressed mainly in jejunum. Therefore, the specific inhibitors can be applied to investigate the transportation mechanism of drug.

Results of infusion experiments in vitro and in situ both showed that the addition of P-gp inhibitors (verapamil hydrochloride and quinidine) and MRP2 inhibitor (probenecid) significantly increased the permeability of TSG in the intestinal epithelium. The involvement of P-gp and MRP2 proteins in the absorption of TSG suggested that TSG may be a substrate of efflux proteins P-gp and MRP2. In other words, in addition to passive transport, there is also an efflux protein-mediated primary active transport mechanism. The intestinal permeability of TSG may, at least partly, be limited by P-gp and MRP2.

In addition to the efflux proteins, metabolic enzymes also play a very important role in the intestinal absorption of drugs [29]. A variety of enzymes related to drug metabolism are expressed in the intestine, such as CYP450, glucuronyltransferase, N-acetyltransferase, glutathione S-transferase and esterase. In the process of intestinal transport, the enzymes of drug metabolism bind to the substrate, forming a metabolite, and the drug absorption is reduced due to the intestinal first pass effect. CYP3A4 is the most important enzyme in the CYP450 family and is involved in intestinal metabolism of approximate 90% drugs. Studies have shown that verapamil hydrochloride can not only inhibit the efflux of P-gp protein, but also inhibit the expression of CYP3A4, which can reduce the metabolism of substrates [30]. Verapamil hydrochloride can promote the absorption of TSG, and we hypothesized that in addition to inhibit P-gp protein, verapamil hydrochloride may also inhibit the metabolism of TSG by CYP3A4, thereby increasing intestinal absorption. Therefore, the impact of drug-metabolizing enzymes on TSG remains to be further studied.

The study combined in vitro Caco-2 cell transport experiments and in situ gastrointestinal perfusion experiments to systematically study the absorption mechanism of TSG in the gastrointestinal tract. Our study found that TSG showed moderate absorption in the Caco-2 cell monolayer model. According to the cell model for drug absorption, it is predicted that its absorption in the intestine should be 20–70%. However, the absorption rate obtained in our study was significantly lower than 20%, and the results of the two models were different. Caco-2 cell monolayer model lacks mucus layer and some metabolic enzymes in intestinal wall, and the barrier characteristics are different from those of small intestinal epithelial cells. It may lead to some differences in transport in vitro and in situ experiment. Therefore, this also reminds us that we need to use a variety of different models to more realistically simulate the internal environment of the body, in order to obtain more realistic and accurate experimental data.

In summary, our study showed that TSG has a low permeability in intestinal epithelial cells. Additionally, we also found that intestinal absorption of TSG involves promoting passive diffusion and may be affected by the efflux transporters P-gp and MRP2. In future studies, we will focus on the effects of intestinal microflora, other transporters and metabolic enzymes on the intestinal absorption mechanism of TSG.

## Conclusions

In this study, we investigated the effects of different concentrations, time, pH and transporter inhibitors on the absorption of TSG in Caco-2 cell model and single-pass intestinal perfusion model. The present study has revealed that TSG is poorly absorbed in the intestines. Both in vitro and in situ experiments showed that the absorption of TSG was saturated with increasing concentration, and it was better absorbed in a weakly acidic environment with a pH of 6.4. In addition, we have also found that the intestinal absorption mechanism of TSG may involve the efflux transporters P-gp and MRP2. As a whole, during the transport process, TSG interacted with P-gp and MRP2, and TSG was not only the substrate of the P-gp and MRP2, but also affected the expression of P-gp and MRP2. In conjunction with results from previous studies along the direction of TSG, these results provided updated information concerning the intestinal absorption process and the possible mechanism of this compound.

## Data Availability

The datasets used and/or analyzed during the current study are available from the corresponding author on reasonable request.

## Abbreviations

C_cum_: Cumulative absorption concentration
DMEM: Dulbecco’s modified eagle medium
FBS: Fetal bovine serum
GAPDH: Glyceraldehyde-3-phosphatedehydrogenase
HBSS: Hank’s banlanced salt solution
HPLC: High performance liquid chromatography
Ka: Absorption rate constant
MRP2: Multidrug resistance-associated protein 2
MTT: Methylthiazolyldiphenyl-tetrazolium bromide
PA: Percentage of absorption
P_app_: Apparent absorption coefficient
PBS: Phosphate buffered saline
P-gp: P-glycoprotein
TEER: Trans-epithelial electrical resistance
TSG: 2,3,5,4′-tetrahydroxystilbence-2-O-β-D-glucoside
